# The Impact of Digital-First Consultations on Workload in General Practice: Modeling Study

**DOI:** 10.2196/18203

**Published:** 2020-06-16

**Authors:** Chris Salisbury, Mairead Murphy, Polly Duncan

**Affiliations:** 1 Centre for Academic Primary Care Department of Population Health Sciences, Bristol Medical School University of Bristol Bristol United Kingdom

**Keywords:** general practice, family practice, electronic consultations, remote consultation, telemedicine, telephone consultation, video, access to health care, health care quality, access, and evaluation

## Abstract

**Background:**

Health services in many countries are promoting digital-first models of access to general practice based on offering online, video, or telephone consultations before a face-to-face consultation. It is claimed that this will improve access for patients and moderate the workload of doctors. However, improved access could also potentially increase doctors’ workload.

**Objective:**

The aim of this study was to explore whether and under what circumstances digital-first access to general practice is likely to decrease or increase general practice workload.

**Methods:**

A process map to delineate primary care access pathways was developed and a model to estimate general practice workload constructed in Microsoft Excel (Microsoft Corp). The model was populated using estimates of key variables obtained from a systematic review of published studies. A MEDLINE search was conducted for studies published in English between January 1, 2000, and September 30, 2019. Included papers provided quantitative data about online, telephone, or video consultations for unselected patients requesting a general practice in-hours consultation for any problem. We excluded studies of general practitioners consulting specialists, consultations not conducted by doctors, and consultations conducted after hours, in secondary care, in specialist services, or for a specific health care problem. Data about the following variables were extracted from the included papers to form the model inputs: the proportion of consultations managed digitally, the proportion of digital consultations completed without a subsequent consultation, the proportion of subsequent consultations conducted by telephone rather than face-to-face, consultation duration, and the proportion of digital consultations that represent new demand. The outcome was general practice workload. The model was used to test the likely impact of different digital-first scenarios, based on the best available evidence and the plausible range of estimates from the published studies. The model allows others to test the impact on workload of varying assumptions about model inputs.

**Results:**

Digital-first approaches are likely to increase general practice workload unless they are shorter, and a higher proportion of patients are managed without a subsequent consultation than observed in most published studies. In our base-case scenarios (based on the best available evidence), digital-first access models using online, telephone, or video consultations are likely to increase general practitioner workload by 25%, 3%, and 31%, respectively. An important determinant of workload is whether the availability of digital-first approaches changes the demand for general practice consultations, but there is little robust evidence to answer this question.

**Conclusions:**

Digital-first approaches to primary care could increase general practice workload unless stringent conditions are met. Justification for these approaches should be based on evidence about the benefits in relation to the costs, rather than assumptions about reductions in workload. Given the potential increase in workload, which in due course could worsen problems of access, these initiatives should be implemented in a staged way alongside careful evaluation.

## Introduction

An increasing number of primary care consultations are being provided under a digital-first model, in which consultations are conducted by telephone, video, email, or online “e-consultation” systems, before offering patients a face-to-face consultation only when necessary. Examples include Doctor On Demand in the United States, Curon in Japan, Ping An Good Doctor in China, and KRY, which operates in several European countries. In England, National Health Service (NHS) policy strongly promotes the use of online consultations [1] and companies such as Babylon GP at Hand, LIVI, and Push Doctor, which offer video consultations free of charge to NHS patients, are expanding rapidly [2]. The introduction of these new access pathways in England will be accelerated by a contract reform framework that will require all general practices to offer online and video consultations by April 2021 and to allow NHS 111 (a national telephone helpline) to directly book face-to-face appointments in local practices [3]. NHS England has recently issued detailed guidance to support the introduction of online consultations [4].

These changes are justified by two main arguments [1-5]. First, they are designed to facilitate quick and convenient access to care by patients, in line with similar changes in how consumers access almost all other services apart from health care. Second, it is argued that the introduction of “online-first” or “telephone-first” models of access will help to manage workload pressures on general practitioners (GPs). However, the twin aims to improve patient access and to manage workload pressures on GPs are likely to be in tension. Whether digital-first models of care decrease or increase general practice workload depends on factors such as the duration of the initial digital consultation and the proportion of these consultations that result in the patient needing a subsequent face-to-face consultation. The impact on general practice workload also depends on whether the demand for consultations is fixed or related to accessibility [6]. At present, many people have difficulty obtaining GP consultations and some may therefore seek help elsewhere or not obtain any professional advice [7]. If quicker and easier access to care means more people contact general practices, this supply-related demand needs to be considered alongside any efficiency gains.

The overall impact on workload in general practice therefore depends on the relationship between several variables. We developed a model to estimate the impact of alternative access pathways on general practice workload and populated the model using a systematic review of studies of digital consultations in primary care. The aim of this study was to inform debate about to what extent, and under what circumstances, digital-first primary care consultations are likely to decrease or increase general practice workload.

## Methods

### Overview

**Figure 1:**
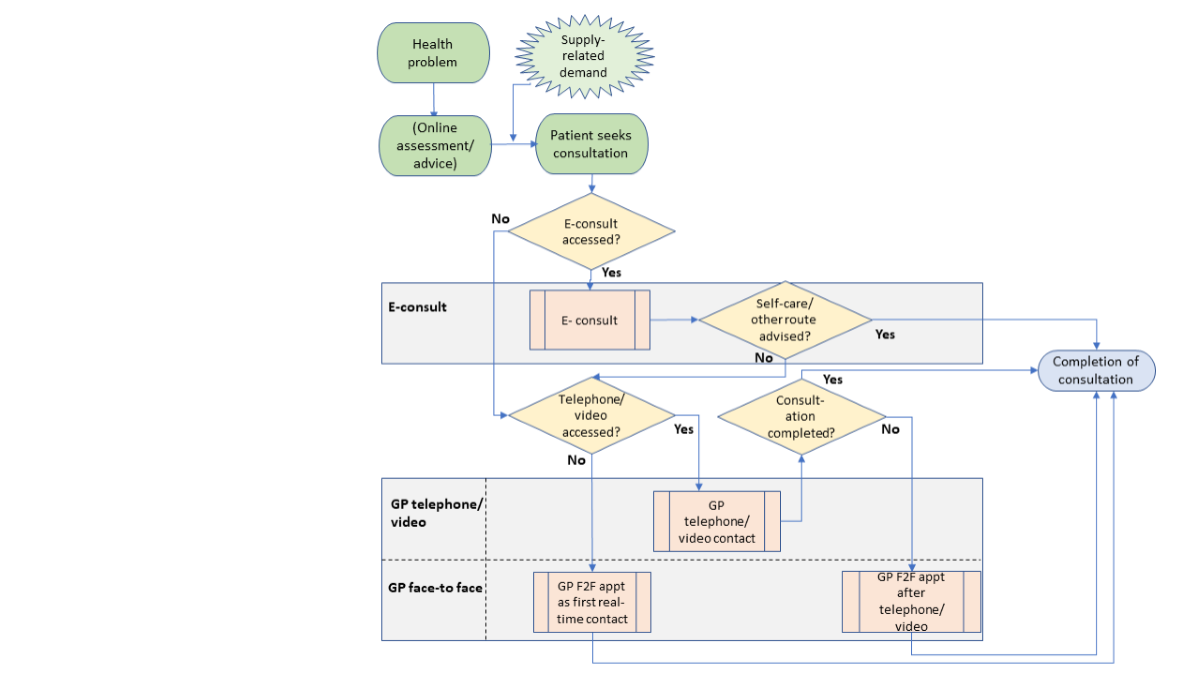
Process map: impact of digital first access pathways on general practitioner (GP) workload. E-consult: online consultation. F2F: face-to-face.

We developed a process map to delineate the access pathway from when patients first seek a general practice consultation through to obtaining definitive assessment and care (Figure 1). Given the interconnectedness of health care systems, any such model is a simplification and must have a defined scope. Our process map begins with a patient having a health problem and considering requesting a GP consultation. They will often seek advice, which may be from family, a pharmacist, or online through an internet search, an automated symptom-checker app, or a patient forum [7]. Our model begins at the point following this, when a patient actively contacts a general practice requesting a consultation. It therefore excludes administrative issues conventionally dealt with by receptionists (for example, repeat prescriptions), consultations usually managed by nurses (for example, vaccinations), and patients who complete an online application but do not seek contact with a GP. The model includes consultations directly necessitated by a previous step (for example, a face-to-face consultation resulting from an online consultation) but ignores follow-up consultations. The model is limited to the impact on GPs, although we recognize that changes in access to GPs can have consequences for administrative and nursing staff and for demand on other parts of the health service.

We populated the workload model using data from a systematic review for 3 scenarios, representing the following 3 access pathways:

- In an online (or e-consultation) first model, patients describe their problem using an electronic form that may involve an automated algorithm or a less structured form. Administrative requests are dealt with by receptionists (excluded by our model). Requests for medical advice are reviewed by a GP who responds with a message or prescription, or a telephone, video, or face-to-face appointment.
- In a telephone-first model, the GP attempts to resolve the problem by telephone if possible, but if not, invites the patient to a face-to-face consultation.
- A video-first model follows a similar pattern, but there may be differences in variables such as the length of the consultation and the proportion of contacts that require a subsequent face-to-face consultation.

### Model

The outcome for the model is percentage change in GP workload using the digital approach compared with GP workload using a conventional approach, in which most patients have face-to-face consultations, but a small proportion have telephone consultations. We created a dynamic spreadsheet model that allowed us to calculate how GP workload changes depending on the values of the key variables in the process map (such as the duration of digital consultations, or supply-related changes in demand). The results from our model are expressed as a percentage, so a result of 10% would mean 10% more hours of work than under conventional care.

The estimates for the conventional approach came from a large and rigorous study of GP consultations in England [8]. The study showed that 86% of consultations were conducted face-to-face and 14% were conducted by telephone, with a mean duration of 9 minutes and 5 minutes, respectively. The estimates for the new digital approaches came from a systematic review, described below. The results from the model illustrate the impact of a central base-case estimate, based on the best available evidence from our systematic review. For each variable in the model, we also considered plausible upper and lower limit estimates based on outlier studies from the review or on our own informed opinion in the absence of evidence. We have made the dynamic model available online, so that readers can test the effect of their own assumptions and estimates [9].

### Systematic Review

To include evidence-based estimates in the model, we conducted a systematic review to identify studies of any design that provided quantitative data about digital consultations in primary care, including consultations by telephone, email, e-consultation systems, or video. The quantitative variables of interest were the proportion of consultations managed digitally, completion rate (digital consultations completed without needing a subsequent GP consultation), the proportion of subsequent consultations managed by telephone rather than face-to-face following an online consultation, the duration of different types of consultation, and any indicators of changes in demand or workload after the introduction of a digital-first model.

A change in the number of requests for health care following improved access is commonly referred to as supply-induced demand [6], which implies that a change in services has caused increased demand. However, in this study we used the more neutral term “supply-related demand” because it could equally represent underlying demand, which becomes visible only once access is improved. We sought to estimate the proportion of digital consultations that represented new supply-related demand based on (per month) the number of contacts of all types after introducing digital consultations, minus the number of contacts before introducing digital consultations, minus the number of duplicate consultations where patients had a face-to-face consultation directly resulting from a digital consultation, divided by the number of digital consultations.

Our focus was on consultations for undifferentiated problems between a patient and a GP; therefore, we excluded studies of communication between health professionals (for example, GPs consulting specialists), after-hours consultations, specialist or secondary care consultations, consultations not conducted by GPs, studies limited to a specific type of health problem, qualitative studies, studies of patient or GP opinion, and systematic reviews that did not provide any new quantitative analyses beyond the already-included papers. Our search strategy included terms for general practice, family practice, or primary care, or papers published in leading primary care journals, combined with a wide range of terms relating to telephone, online, digital, or video consultations. We included papers identified through the bibliographies of other papers. Multimedia Appendix 1 shows the search strategy, which was conducted in MEDLINE. We restricted searches to papers published since January 1, 2000, in English, and in developed countries to focus on papers of current relevance to the United Kingdom and other similar health care systems. We did not attempt to grade the quality of the studies or assess the risk of bias. The searches were updated to September 30, 2019. CS and PD reviewed the titles and abstracts of papers independently, retaining any papers that were potentially relevant. They then independently reviewed the full text of these papers to identify those that met the inclusion criteria. CS extracted quantitative data about the variables of interest, and this was checked by PD. Disagreements between reviewers were resolved by discussion involving the third author, MM.

## Results

The systematic review identified 1246 papers, of which 90 were judged to be potentially relevant based on their titles and abstracts (Multimedia Appendix 2). Of these, 29 papers provided data of relevance to this study (Multimedia Appendix 3) [8,10-37]. Table 1 shows the estimates for the variables included in the workload model.

Based on our workload model, the final row in Table 1 shows workload in general practice using our central estimates for each scenario, compared with a conventional pathway based predominantly on face-to-face consultations.

**Figure 2:**
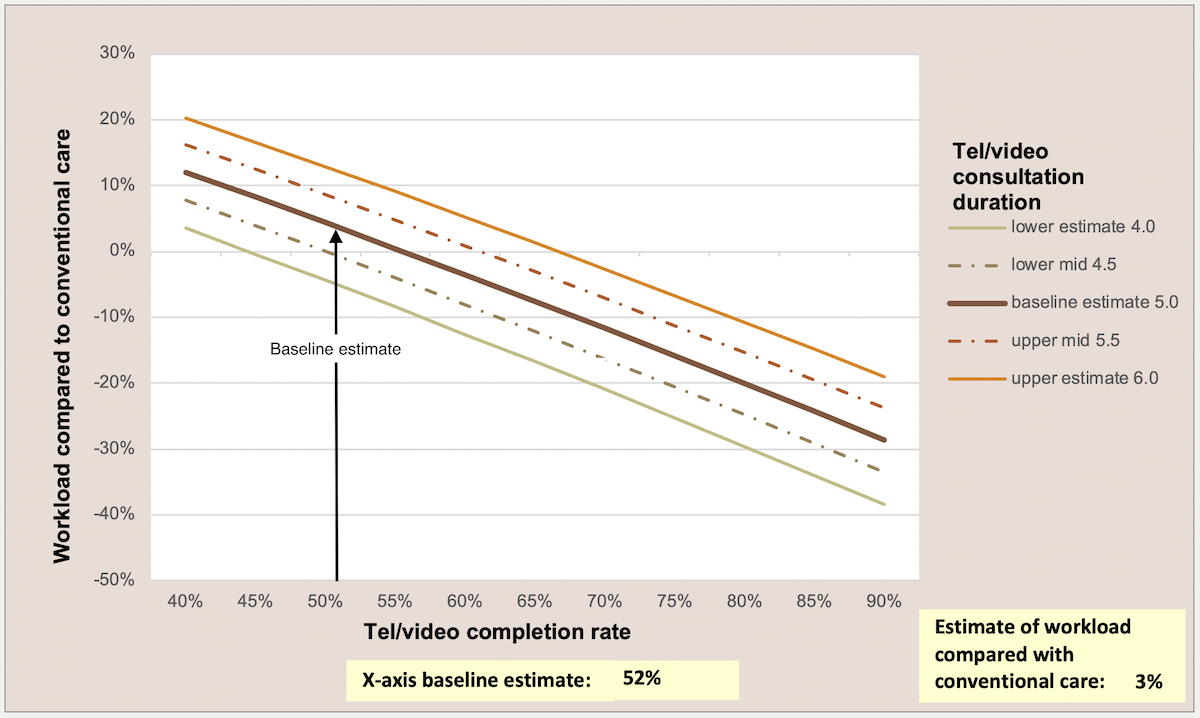
Impact of telephone consultations on general practitioner workload: varying telephone completion rate and call duration.

**Figure 3:**
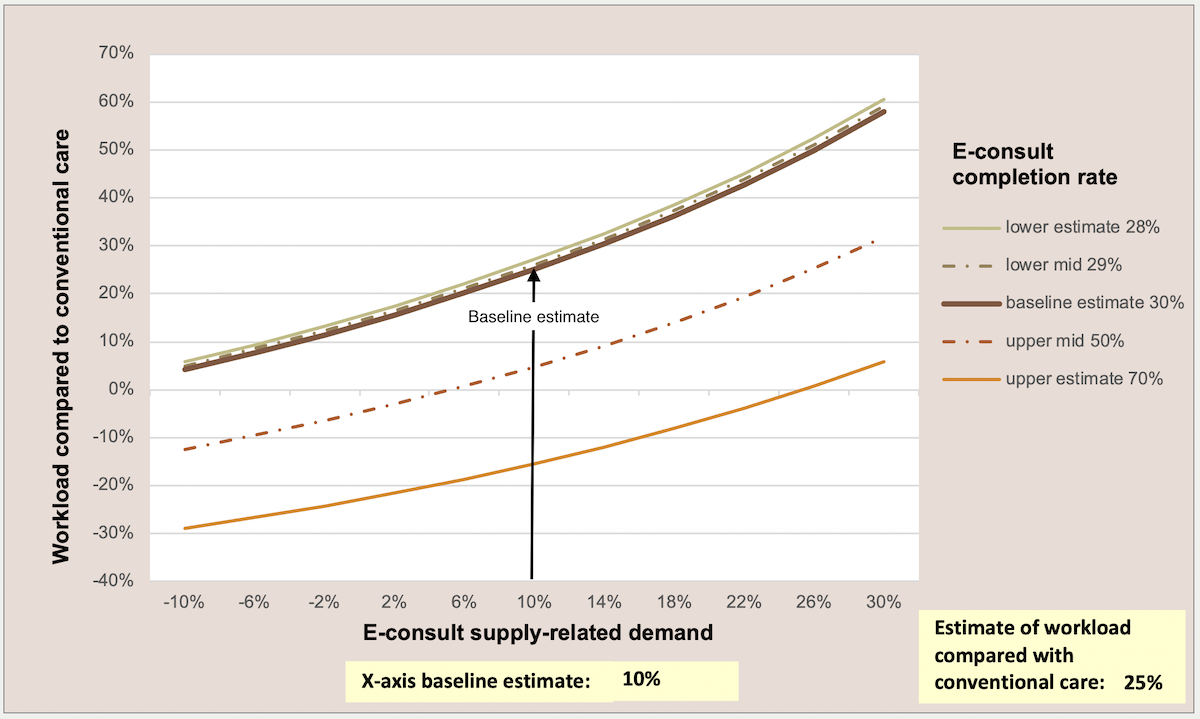
Impact of e-consultations on general practitioner workload: varying supply-related demand and e-consultation completion rate. E-consult: online consultation.

The dynamic model makes it possible to test the sensitivity of the model to different assumptions by graphically showing the impact on workload (y-axis) of changing any 2 variables simultaneously (x-axis and legend). In Figures 2 and 3, we show 2 different scenarios as examples; readers can use the model to test their own scenarios [9]. Figure 2 shows that if the average duration of a telephone call is 5 minutes, a telephone-first approach has the potential to reduce workload if at least 55% of telephone consultations are completed without needing a subsequent face-to-face consultation, assuming no supply-related increase in demand. Figure 3 demonstrates that an online-first approach could reduce GP workload only if there is minimal increased demand (<4% of online contacts represent new demand) and about 50% of all requests are resolved in one online contact, but it has the potential to substantially increase workload if there is any supply-related increase in demand. However, 2 recent UK studies suggest that only about 30% of online consultation requests are resolved entirely online [13,17], and several studies suggest that there could be a substantial inflation in demand [14,16,26,31].

**Table 1:** Alternatives to face-to-face consultation: default values for variables in workload model. All values can be altered in the interactive model to test different scenarios and assumptions. Values without citations are authors’ estimates.

Variable		E-consultation^a^			Telephone			Video		
		Base case	Lower value	Upper value	Base case	Lower value	Upper value	Base case	Lower value	Upper value
Access rate: Consultations initially requested in this way^b^ (%)		90^b^	0.01 [17]	100	93 [12]	10 [20]	100	90^b^	50	100
**Completion rate**										
	Digital consultations completed without needing a subsequent consultation (%)	30 [17]	28 [13]	70 [33]^c^	52 [25,29]	40	90 [20]	65	50	83 [10]
	Of those having a real-time consultation after e-consultation, consultations having phone rather than face-to-face consultation (%)	46 [17]	20	90	N/A^d^	N/A	N/A	N/A	N/A	N/A
Average time: Average time spent by GP^e^ on this type of consultation (minutes)		4	3	5 [17]	5 [8]	4 [11,15]	6 [29]	9^f^	6 [21]	15 [34]
Supply-related demand: Alternative form consultations that are new demand (%)		10	–10	30	0 [29]	–10	30	0	–10	30
Total workload resource compared with conventional care, using base case assumptions (%)		25	N/A	N/A	3	N/A	N/A	31	N/A	N/A

^a^E-consultation: online consultation.

^b^At present, usage of e-consultation and video consultation in the United Kingdom is generally very low, so the impact is minimal. For the base cases, we have modeled a scenario in which the use of these alternatives is usual.

^c^Penza et al report a 66% completion rate [33]. Completion rates of 70% are claimed by eConsult, cited by Marshall et al [2]. Longman reports similar experiences in practices using askmyGP [38].

^d^N/A: Not applicable.

^e^GP: general practitioner.

^f^Assumed to be similar to conventional face-to-face care.

## Discussion

### Principal Results

Current initiatives to improve access and reduce GP workload through digital-first approaches could have benefits for patients and GPs or could have entirely the opposite effect to that intended. Based on current evidence, these approaches are at least as likely to increase as to decrease workload pressure on general practice. There is potential to reduce general practice workload, but only under stringent conditions, whereby the initial assessment is short and a high proportion of contacts are managed without needing a subsequent face-to-face review. Under almost all scenarios, even modest increases in demand related to improved accessibility would lead to increases in workload.

### Comparison With Prior Work

The estimates used to populate this model are based on the best available published evidence. Companies providing digital-first consultation systems claim that general practices using their systems achieve impressive improvements in access and reductions in workload [38,39], and anecdotal reports from some early adopter practices suggest that these benefits can be achieved [4,40]. However, research studies involving larger numbers of practices show that this is not necessarily generalizable [12,29], and UK health services that have introduced digital-first approaches have reported that they do not appear to save GP time [13,41-43]. Furthermore, 2 studies have suggested that telephone-first approaches that lead to reductions in consultations on the same day are compensated for by increased consultations over the next 28 days [11,28]. Evidence from other countries relates to consultation rates or costs rather than workload and provides conflicting findings, with some studies reporting that offering digital consultations leads to increases (including the online consultations themselves) [14,16,26,30,31], while other studies report reductions [18,22,37]. This implies that it is important to understand how and why digital-first approaches are successful in some circumstances but not others. This study helps to inform this debate.

### Strengths and Limitations

To ensure comparability between different access pathways, the denominator for our model was patients who decide to seek a consultation with a GP, rather than all patients who use an automated symptom-checker or triage app. About one-third of people look for information online about their symptoms in a conventional consultation system [7], so the use of symptom-checker apps may be partly substituting for this use rather than replacing consultations. The possibility that the availability of an online symptom-checker or triage app could decrease or increase the number of requests for contact with a GP is taken into account using the supply-related demand parameter in our model. Although some providers of online consultation services report that a high proportion of contacts can be managed entirely online [38,39], it is important to consider the denominator, since some of these contacts might not have been made at all or would not have led to a consultation under conventional care. These effects can be considered by comparing the total number of online, telephone, and face-to-face contacts with the number of consultations under a previous conventional approach (see formula in “Systematic Review” in the Methods section).

Some of the estimates used in this model could be challenged because they depend on the context in which the access pathway is introduced. For example, some of the estimates in the model come from the ESTEEM trial of telephone triage of requests for same-day consultations rather than all consultations [11,12]. Second, some clinicians suggest that the duration of a face-to-face consultation might be shorter after a prior online or telephone assessment than under conventional care [19] (although the evidence suggests this is not the case) [23,29]. Third, the proportion of consultations that can be successfully completed online or by telephone might be higher when patients are given the choice to consult in this way, rather than in systems where all patients have to go through this step before accessing a face-to-face consultation. A strength of this study is that by making our model freely available, anyone can explore how workload varies under different assumptions.

The need to limit the scope of the workload model means that it does not consider the effect on other services. The availability of digital consultations could be efficient if it reduces consultations in hospital emergency departments, but the evidence so far provides little support for this hypothesis [12,29,34,44]. The use of digital-first approaches could reduce GP workload by directing patients needing face-to-face care to other primary care professionals, such as nurses and pharmacists. However, we were unable to include this in the model because of a lack of evidence about how much this delegation occurs (compared with the extent to which receptionists direct patients to these professionals under conventional appointment systems) and the proportion of patients that would need to be transferred back to a GP after a nurse or pharmacist consultation. It will be increasingly important to consider the impact on nonmedical workloads in general practice as roles and responsibilities evolve. It is important to note that although delegation to other staff may reduce GP workload, it is not necessarily more efficient for the health care service overall [45].

Finally, we recognize that this study was designed from the perspective of the NHS in the United Kingdom, and usual care is different in other countries. However, by making the workload model freely available, including allowing changes to estimates such as the duration of face-to-face consultations under usual care, we hope that our model will be useful in various settings.

### Implications for Clinicians and Policy Makers

If initiatives to improve access to care do lead to increased GP workload, this is not necessarily an argument against them. Ensuring good access to health care is a core purpose of primary care, and additional consultations might represent a response to previously unmet need. However, these initiatives should be justified on the grounds of benefits to patients rather than claims about reductions in GP workload [46]. As with most medical interventions, the key issue is whether the additional benefits are justified in relation to any extra costs. Furthermore, our analysis shows that assumptions about efficiency savings may be misplaced and general practice may need more resources to implement digital-first pathways.

It is important to consider how the benefits of different access pathways are felt by different segments of the population. Digital consultations are predominantly used by patients in the 20-44 age group [17,34], which is a group with generally fewer health care needs. If improving access for them requires more GP time, this will decrease rather than increase the time available for patients with more complex problems, as well as decreasing access for those without internet access. To reduce the potential for worsening health inequalities, it is important to prioritize the use of technology to improve access for the groups of patients with the greatest health care needs, such as older adults, carers, and people with disabilities.

Digital-first access models may have other potential advantages and disadvantages. Improved access could help reverse the decline in public satisfaction with NHS general practice [47] and help avoid inappropriate use of expensive hospital care [3]. Triage systems may offer GPs a greater sense of control over their working day [4]. Technologies to allow GPs to work from home could expand the workforce by unlocking the potential contribution of doctors who cannot work fixed hours in conventional settings [34]. On the other hand, a shift in working patterns toward significant amounts of time spent consulting online or by telephone could lead more GPs to leave the workforce than to join it.

Apart from the impact on workload, there are very important unknowns about the quality and safety of alternatives to face-to-face consultations, as well as the acceptability of these access pathways to different patient groups [2]. These questions should be a priority for research.

### Conclusions

This study has highlighted that efficiency gains or losses from the use of digital-first access pathways are finely balanced, and the main impact on workload will be determined by whether these pathways change demand. Digital-first services could increase demand through improved supply or surfacing previously unmet need, or could reduce demand by encouraging patients to self-care or use other services. This is, therefore, an issue of critical importance, but about which we currently have the least evidence. It may take several years for these effects to become manifest. Given that it will be difficult to lower expectations and demand after these have been raised, this suggests the need for careful and staged implementation alongside evaluation rather than universal implementation of digital-first access pathways as soon as possible, as advocated by current UK policy [3].

## Appendix

Multimedia Appendix 1 Search strategy for literature in MEDLINE (Ovid SP).

Multimedia Appendix 2 PRISMA Flow Diagram.

Multimedia Appendix 3 Data about model inputs from relevant papers.

## Abbreviations

GP: general practitioner
