# Supplementary material for: The Impact of Digital-First Consultations on Workload in General Practice: Modeling Study
Source: J Med Internet Res. 2020 Jun 16;22(6):e18203. doi: 10.2196/18203 (PMC7327596; doi:10.2196/18203)
Supplement: Multimedia Appendix 1 [file jmir_v22i6e18203_app1.docx]

## Appendix 1. Search strategy for literature in MEDLINE (Ovid SP)

1. primary care.mp. or Primary Health Care/

2. family practi*.mp. or Family Practice/

3. general practi*.mp. or General Practice/

4. family medicine.mp.

5. primary health care.mp.

6. 1 or 2 or 3 or 4 or 5

7. "british journal of general practice".jn.

8. "annals of family medicine".jn.

9. family practice.jn.

10. "european journal of general practice".jn.

11. bmc family practice.jn.

12. ("journal of the american board of family medicine jabfm" or "journal of the american board of family practice").jn.

13. canadian family physician.jn.

14. american family physician.jn.

15. "scandinavian journal of primary health care".jn.

16. australian family physician.jn.

17. 7 or 8 or 9 or 10 or 11 or 12 or 13 or 14 or 15 or 16

18. 6 or 17

19. ((telephon* or phone or online or e?mail or internet or digital or remote or video or Skype or Facetime or electronic or web or web-based or e-consult or econsult) adj (triage or consultation$ or visit$ or assessment$ or advice or messag$)).mp.

20. 18 and 19

21. 20

22. limit 21 to (english language and yr="2000 -Current")
