# Supplementary material for: The Impact of Digital-First Consultations on Workload in General Practice: Modeling Study
Source: J Med Internet Res. 2020 Jun 16;22(6):e18203. doi: 10.2196/18203 (PMC7327596; doi:10.2196/18203)
Supplement: Multimedia Appendix 2 [file jmir_v22i6e18203_app2.docx]

## Appendix 2 PRISMA Flow Diagram

Records identified through database searching (n = 1240)

## Screening

## Included

## Eligibility

## Identification

Additional records identified through other sources (n = 6)

Records after duplicates removed
(n = 1246)

Records screened
(n = 1246)

Records excluded
(n =1156)

Full-text articles assessed for eligibility
(n = 90)

Full-text articles excluded:

Not patient-GP consultation (n = 17)

Specialist topic or service (n = 1)

No data about variables of interest (n = 37)

Systematic review with no new data (n = 6)

Studies included in review
(n = 29)
