# Supplementary material for: The Impact of Digital-First Consultations on Workload in General Practice: Modeling Study
Source: J Med Internet Res. 2020 Jun 16;22(6):e18203. doi: 10.2196/18203 (PMC7327596; doi:10.2196/18203)
Supplement: Multimedia Appendix 3 [file jmir_v22i6e18203_app3.docx]

**Appendix 3 Data about model inputs from relevant papers**

Model inputs were the proportion of consultations managed digitally, the completion rate (proportion of digital consultations completed without a subsequent face-to-face consultation), duration of consultation, and supply-related changes in demand after introduction of a digital-first model. We also recorded papers which directly or indirectly provided data about our outcome of interest, GP workload.

| First author, Year | Digital-first approach | Country | Estimate | Notes |
| --- | --- | --- | --- | --- |
| Proportion of consultations managed digitally | | | |  |
| Edwards, 2017 | Online | UK | 0.002% | See page 4, col 2, end first paragraph |
| Huygens, 2018 | Online | Netherlands | 0.70% | See page 4, dataset 1, end of first paragraph, 2014 data. Refers to email consultations |
| Carter, 2018 | Online | UK | 5% | See table 3. e-consult rate varies in different practices; maximum of 5% in practice 6 |
| Campbell, 2014 | Telephone | UK | 93% | See Figure 1 GP Triage arm |
| Gonzalez, 2018 | Telephone | Spain | 10% | In a system where people could log in and book a phone or F2F appt, 10.1% chose phone in 2015 (see 'efficiency of telephone consultation') |
| Completion rate |  |  |  |  |
| Edwards, 2017 | Online | UK | 30% | 70.2% of e-consults resulted in phone or F2F. Of these 45.7% (32.1/70.2) were by phone. See table 3. NB same data as Farr et al (2018) |
| Penza, 2018 | Online | USA | 66% | Only 34% reconsulted within 30 days, so at least 66% managed online only initially |
| Carter, 2018 | Online | UK | 28% | See 'CRFs completed by GPs' second paragraph; in 72% of e-consults the GP suggested a face-to-face or phone consultation was needed |
| Farr, 2018 | Online | UK | 30% | 70.2% of e-consults resulted in phone or F2F. Of these 45.7% (32.1/70.2) were by phone. See table 2. NB same data as Edwards et al (2017) |
| Jiwa, 2002 | Telephone | UK | 53% | Calculated from Table 1, including advice only, prescription offered, appointment with nurse |
| Campbell, 2015 | Telephone | UK | 64% | See Table 15, p48. Of 5171 people having GP phone triage, 1850 (35.8%) had GP F2F on same day. NB this may be an overestimate of true completion rate because additional patients had nurse F2F consultations or other actions, and some may have had GP F2F consultation on the next day. |
| Newbould, 2017 | Telephone | UK | 52% | Based on survey responses in table 4, 48% had F2F or phone appointment with GP after phone triage |
| Gonzalez, 2018 | Telephone | Spain | 90% | If patients chose phone consultation, only 10.3% needed a subsequent F2F (2015 data) |
| Brunett, 2015 | Video | USA | 83% | Uncertain inclusion, since video-consultations only available for a limited specified list of 16 medical problems. Of people choosing a video consultation only 17.2% were called in for F2F consultation |
| Duration |  |  |  |  |
| Edwards, 2017 | Online | UK | 5 mins | NB. estimated by practice staff, not directly measured |
| Peleg, 2001 | Telephone | Israel | 5.8 mins | Very small study, diary of one GP, most calls were from friends or colleagues. |
| de Groot, 2002 | Telephone | Netherlands | 4.2 mins | Based on a call-back system to replace responding to incoming calls. Data collected 1997 |
| Richards, 2002 | Telephone | UK | 4.7 mins | GP telephone triage estimate from table 3 |
| McKinstry, 2002 | Telephone | UK | 5.2 mins | F2F appointment was 8.2 minutes. NB. This paper also reports an increase in the no. of subsequent surgery contacts in the following two weeks. Consistent with larger and more recent Esteem trial showing reduction in workload on the same day compensated for by additional workload in subsequent days |
| McKinstry, 2010 | Telephone | UK | 4.6 mins | See Table 3. F2F consultation was 9.7 mins |
| Campbell, 2015 | Telephone | UK | 4.0 mins | F2F consultations were 12.4mins after GP triage, 9.8 mins in usual care |
| Hobbs, 2016 | Telephone | UK | 5.4 mins | GP F2F had mean duration of 9.22 mins |
| Holt, 2016 | Telephone | UK | 4.0 mins | Same study as Campbell et al 2015. F2F consultations were 12.4mins after GP triage, 9.8 mins in usual care |
| Edwards, 2017 | Telephone | UK | 7.6 mins | See 'cost of e-consultations’ but based on phone calls after an e-consult, so probably filtering out simple cases. Also shows that F2F consultations were 14.5 mins after econsult i.e. longer than national average |
| Stevens, 2017 | Telephone | UK | 5.3 mins | Same data as Hobbs et al. 2016. Also shows F2F duration of 9.24 mins |
| Newbould, 2017 | Telephone | UK | 6.2 mins | From data supplement 3, based on duration after introduction of GP access system |
| Hammersley, 2019 | Telephone | UK | 5.6 mins | See Table 4 |
| Hammersley, 2019 | Video | UK | 5.9 mins | See Table 4. NB Not controlled for age. Video users were younger. This study was just of follow-up consultations, not new problems. |
| Quigley, 2019 | Video | UK | 15 mins | Estimate based on consultation rate of 4 consultations per hour |
| Supply-related demand* | | |  |  |
| Katz, 2003 | Online | USA | Increase | Most e-consults represent new demand. Based on table 2, period 5, difference between calls in intervention and control groups. But difficult to apply, since only refers to change in telephone calls, doesn't report details of face-to-face consultations. |
| Bergmo, 2005 | Online | Norway | Reduction | Small RCT in one practice. Total no. of consultations (including online) was less after offering online. i.e. online not only replaced F2F consultations, it reduced total number of consultations of any type. Numbers also dropped (but less) in control group. Probably regression to the mean, since patients were recruited from waiting room, so likely to be consulters. |
| Zhou, 2007 | Online | USA | Unclear | Doesn't provide comparable data to this study. Suggests that online consultations lead to lower workload through fewer F2F and telephone consultations but does not provide any details of the number of online consultations. Study likely to have been affected by regression to the mean, since cases recruited when they used online and no adjustment for prior health service utilisation in comparing cases and controls. |
| Chen, 2009 | Online | USA | Increase | Increase & substitution of consultation types. Big reductions in F2F and telephone consultations, even bigger increase in online consultations. Overall consultation rates increased by 8%. This was interpreted as being more efficient on the assumption that online consultations are quicker and cheaper, but these were not measured. |
| Palen, 2012 | Online | USA | Increase | Access to online records including ability to contact GP online was associated with 26% increase in office visits (0.7/2.7) and 8% increase in phone calls (0.3/3.9). Similar to Zhou et al (2007) (both from Kaiser) but this one is more recent, larger and uses more sophisticated matching of cases and controls, including matching on prior utilisation. Reaches opposite conclusion from the earlier paper. Neither paper takes account of the number of online consultations themselves. |
| North, 2014 | Online | USA | Increase | Reports no significant difference in no. of office visits after introducing online portal but does not mention the number of online consultations themselves, implying that most online contacts represent additional consultations. |
| Dexter, 2016 | Online | USA | Increase | Authors hypothesised a negative correlation between online and phone calls, if online reduced phone calls. In fact, they observed a positive correlation in 2 clinics and no relationship in 2 clinics, suggesting that most online contacts represent additional consultations. |
| Newbould, 2017 | Telephone | UK | 0.20% | Based on ((21.8-16.5 - (12.2*0.44))*100) / (12.2 - 3.0). (See formula shown in the methods section of the current paper). Data come from first section of results. Survey (table 4) shows that 44% of patients phoned were called for a F2F GP consultation. |
| GP workload* |  |  |  |  |
| Edwards, 2017 | Online | UK | Increase | Based on 10% increase in practice cost (not just GP workload) compared with national average. |
| Ekman, 2018 | Online | Sweden | Unclear | A modelling study of costs rather than workload. Suggests substantial reductions from online consultations but takes no account of proportion of online consultations which lead to a subsequent F2F consultation, nor the possibility of supply-related demand. |
| Campbell, 2014 | Telephone | UK | -2.6% | No significant difference in costs of GP time in primary analysis of consultations over 28 days (£64.67 GP phone triage, £66.39 usual care). See table 2. Same study as Campbell et al, 2015 |
| Campbell, 2015 | Telephone | UK | -18% (on same day only) | This study analyses costs, which is equivalent to workload when only GPs are considered. Table 31 shows costs on same day only (secondary analysis). Considering GP costs only, (not patients called in for subsequent day, or nurse consultations) GP costs £39.67 in usual care, £32.55 in GP triage. But in main analysis (table 28; all consultations over 28 days) there was no significant difference in GP costs (£66.39 usual care, £64.67 GP triage) so savings on index day were mostly deferred consultations to subsequent days |

* We have not included numeric estimates for supply-related demand or for some studies of workload because these were impossible to calculate in a comparable way across different studies, and most studies do not include details of all consultation types.
